# Exploring protein adenylyltransferase as a therapeutic target for combating ESKAPE pathogens in hospital-acquired infections

**DOI:** 10.1007/s11033-025-10735-5

**Published:** 2025-07-07

**Authors:** Reabetswe Maake, Sarah Otun, Ikechukwu Achilonu

**Affiliations:** Protein Structure‑Function and Research Laboratory, Faculty of Science, School of Molecular and Cell Biology, University of the Witwatersrand, Braamfontein, Johannesburg, 2050 South Africa

**Keywords:** Antimicrobial resistance, ESKAPE pathogens, Hospital-acquired infections, Protein adenylyltransferase, Glutaredoxin, AMPylation, Glutathione, Glutathionylation, Deglutathionylation

## Abstract

**Introduction:**

In the face of increasing antimicrobial resistance, ESKAPE pathogens—***Enterococcus faecium*, *Staphylococcus aureus*, *Klebsiella pneumoniae*, *Acinetobacter baumannii*, *Pseudomonas aeruginosa, and Enterobacter species***—pose a significant threat to public health, particularly in nosocomial settings.

**Areas covered:**

This review explores the potential of targeting protein adenylyltransferase (PrAT) as a therapeutic strategy against these multidrug-resistant bacteria. We discuss the mechanisms of PrAT activity, its involvement in reduction–oxidation (redox) homeostasis, and the rationale for its potential as a drug target against ESKAPE pathogens.

**Expert opinion:**

PrAT plays an essential role in sustaining the bacterium’s redox homeostasis, a vital aspect of bacterial survival, by interacting with glutaredoxin (Grx). Future research should focus on elucidating the specific role of PrAT in ESKAPE pathogens, with an emphasis on studying the enzyme’s function and designing targeted inhibitors. This review underscores the importance of continued investigation into PrAT in ESKAPE pathogens as a critical step in addressing the challenges of antimicrobial resistance in clinical practice.

## Introduction

Nosocomial infections synonymously known as Hospital-acquired infections (HAIs) pose a significant economic burden on healthcare institutions. In 2022, these infections in sub-Saharan Africa resulted in an estimated cost of $13 billion [1]. Beyond the financial implications, nosocomial infections contribute to increased morbidity and mortality rates in healthcare settings [2]. The prolonged hospital stays necessitated by these infections lead to the overuse of antibiotics, which fuels the rise of multidrug-resistant (MDR) pathogens. The bacterial pathogens responsible for HAIs were grouped and termed ESKAPE pathogens [3]. These pathogens can evade the effects of several antibiotics, making them MDR. ESKAPE pathogens employ multiple mechanisms to resist current antimicrobial agents. These mechanisms include decreasing the intracellular concentration of antibiotics, modifying the drug target, inhibiting the antimicrobial agent, and synthesising biofilms [4]. It is estimated that more than 70% of bacteria associated with HAIs are resistant to at least one of the antimicrobial agents used to treat them [5]. The available approaches to combat MDR bacteria include creating new antibiotics, combining therapeutic mechanisms or agents, bacteriophage therapy, and peptides against microbes [6, 7].

Although producing new antibiotics would be an evident approach to combating MDR bacteria, one drawback of this approach is that MDR bacteria can gain resistance to newly developed antibiotics. Hence, combination therapy would attenuate the outcome of producing new antibiotics. Combination therapy is the use of at least one method of treatment against a single condition [8]. This approach has proven to be effective against bacterial infections caused by MDR bacteria [9]. This form of therapy would involve using antibiotics that would attack different pathways [6]. Another effective approach would be the use of Bacteriophages. Bacteriophages are viruses that infect bacteria. Bacteriophage therapy entails using either the virus, an engineered form of the virus, or pure virus proteins that are key in lysing the bacteria [10]. Additionally, antimicrobial peptides are naturally occurring small peptides with antibacterial functions and can be employed as antimicrobial agents [11]. Lastly, one proposed approach, which is the focus of this review paper, is to disrupt redox reactions, thereby hindering bacterial survival [12]. Redox homeostasis is achieved when the cell balances reduction and oxidation reactions. This balance modulates cellular responses [13]. The increase in oxygen in the atmosphere due to photosynthetic cyanobacteria bore aerobic metabolism [14]. Aerobic metabolism, cell proliferation and apoptosis are all consequences of redox reactions [13]. In addition to aerobic metabolism the oxygenated atmosphere brought reactive oxygen species (ROS) [15], which are toxic to cells, and when the concentration is high, they induce oxidative stress that is mitigated by antioxidants [15, 16]. Antioxidants prevent oxidation and thus prevent the formation of ROS [16]. Selenoprotein-O (SelO) is a selenoprotein containing selenocysteine (Sec) [17]. Sec is an amino acid-like cysteine, but instead of sulfur, it has selenium on its side chain [18]. Selenoproteins are associated with redox reactions. [17]. Protein kinases aid in the transfer specifically of the γ-phosphate from ATP to substrate containing serine, threonine or tyrosine [19, 20], and the hydroxyl group of the amino acids are targeted for the nucleophilic attack [21]. Thus, pseudokinases are enzymes with the same fold as the kinase but lack amino acids or motifs key in the catalysis of kinases [22]. Protein kinases and pseudokinases are vital in cell processes such as cell signalling [23, 24].

The research gap lies in developing novel therapeutic agents beyond those currently available that can effectively combat MDR bacteria associated with HAIs. A recent study has linked a pseudokinase, known as protein adenylyltransferase (PrAT), to redox homeostasis in bacteria through its interaction with glutaredoxin (Grx) [25]. Grx is a small peptide that reduces thiol groups in glutathione [26]. Glutathione, composed of glycine, cysteine, and aspartate, functions as an antioxidant by neutralising ROS [27]. Thus, PrAT plays a crucial role in maintaining antioxidant defence.

Therefore, this paper aims to shed light on the pivotal role of PrAT and its association with redox homeostasis. It will also delve into the broader context of PrAT in bacteria, its interaction with Grx, and the potential role of targeting this pathway in treating MDR bacteria associated with HAIs. By disrupting critical pathways required for bacterial survival and drug resistance, this research will pave the way for new therapeutic approaches to combat the rising threat of HAIs caused by ESKAPE pathogens, inspiring new research, and innovation in this field.

## Nosocomial infections: a persistent challenge in patient care

HAIs are infections that arise after a patient’s admission to a healthcare facility, typically appearing at least two days post-admission and are not present upon admission to the healthcare facility [28]. These infections pose a significant burden on healthcare systems globally, correlating with increased rates of morbidity and mortality. In developing countries, HAIs are notably more prevalent, with a rate of 15.5%, surpassing that of developed countries by 10.5% [29, 30]. In sub-Saharan Africa, HAI prevalence is estimated between 7–28% [31]. Critical care practices, especially the use of medical devices like catheters and ventilators, have been implicated in the transmission of HAIs [32]. The most common HAIs include central line-associated bloodstream infections (CLABSIs), catheter-associated urinary tract infections (CAUTIs), ventilator-associated pneumonia (VAP), and nosocomial surgical site infections (SSIs) [28]. Mortality rates for these infections range from 2.3–14.4% [33]. Vulnerable groups, such as intensive care unit (ICU) patients, neonates, and immunocompromised individuals, are disproportionately affected by HAIs [34, 35]. Over half of ICU patients in developed countries contract an HAI during their stay, with more than 20% of neonates in developing countries similarly affected [36, 37]. Among ICU patients, CAUTIs, SSIs, and VAP are the most prevalent, while CLABSIs are more frequent in neonatal intensive care units [38, 39]. MDR bacteria are the leading cause of HAIs, particularly in cases of nosocomial pneumonia, such as VAP [28].

## ESKAPE pathogens: a major concern in hospital settings

MDR bacteria account for over 15% of HAIs globally [40]. The bacterial pathogens that predominantly cause HAIs and exhibit multidrug resistance are classified as ESKAPE pathogens for their ability to evade the effects of antimicrobial agents [3].

*E. faecium* belongs to the genus *Enterococcus,* discovered in 1899. This genus is responsible for up to 20% of urinary tract infections (UTIs) in healthcare settings. *E. faecium* uses several resistance mechanisms, including antibiotic modification, inhibition of antimicrobial target activity, and biofilm production [41]. Vancomycin is a glycopeptide antibiotic. HAIs associated with vancomycin-resistant *Enterococci* (VRE) are due to *E. faecium* [42]*.* Vancomycin-resistance *E. faecium* strains are responsible for HAIs such as CLABSIs and CAUTIs [43]. In addition to vancomycin resistance, *E. faecium* is resistant to β-lactam and aminoglycoside antibiotics [44].

Similarly, *S. aureus* colonises the human skin but becomes virulent once it enters the bloodstream. It is responsible for bloodstream infections and hospital-associated pneumonia [45]. The bacterium is resistant to methicillin, a β-lactam antibiotic, leading to the development of methicillin-resistant *S. aureus* (MRSA) strains [46]. MRSA strains are also resistant to all β-lactam antibiotics, aminoglycosides, and vancomycin. MRSA evades the effects of antimicrobial agents by altering the drug target. Fluoroquinolones remain effective antibiotics against gram-positive bacteria, including MRSA [47].

Furthermore, *K. pneumoniae* belongs to the *Enterobacteriaceae* family, first isolated in 1882. It populates the mucosal surfaces of the gastrointestinal tract and the oropharynx [48, 49]. The bacterium is one of the leading causes of HAIs, predominantly nosocomial UTIs, bloodstream infections and pneumonia, and it is accountable for up to 8% of HAIs [50, 51]. *K. pneumoniae* forms biofilms on medical devices and produces strains containing an extended spectrum of β-lactamases (ESBLs) or carbapenemases [48]. The ESBLs, including carbapenemases, are a group of enzymes that can act on a wide range of β-lactam antibiotics such as penicillin, cephalosporins and carbapenems. This means bacteria with these enzymes resist a wide range of β-lactam antibiotics.

*A. baumannii* populates the skin and mucosal surfaces exposed to the environment and has been shown to produce biofilm to promote its survival on medical equipment [52]. Additionally, the bacterium employs the expression of porins or efflux pumps to reduce intracellular concentration of antibiotics, modify the drug target and decrease the antibiotic’s activity as a resistance mechanism against antimicrobial agents [53]. Nosocomial UTIs and VAPs are among the HAIs caused by *A. baumannii* [54]. Carbapenem-resistant strains of *A. baumannii* and strains resistant to penicillin have been isolated [52]. *P. aeruginosa* causes CAUTIs and VAPs in healthcare institutions [55]. It uses natural resistance and efflux pumps to regulate the permeability of hydrophilic substances, such as antibiotics, across the cell membrane [56]. Aminoglycosides and β-lactam antibiotics are some antibiotics the bacterium is resistant to [57].

*Enterobacter species* colonise humans’ skin and gastrointestinal tract and are responsible for healthcare-associated SSIs and UTIs [58]. The predominant resistance mechanism in these bacteria is the production of β-lactamase, which renders them resistant to β-lactam antibiotics [59]. These species have additional resistance to fluoroquinolones and aminoglycoside antibiotics [60]. The current treatment for gram-negative MDR bacteria is colistin, but resistant strains have emerged [61]. The issue is that the pool of effective and available therapeutic agents against MDR bacteria is reducing. The potential therapeutic approach against MDR bacteria would be targeting metabolic processes central to bacterial survival. One possible approach is to target the reduction–oxidation homeostasis in bacteria.

## Overview of protein adenylyltransferase: structure, and function

### Molecular mechanism of protein adenylyltransferase (PrAT)

Protein adenylyltransferase transfers an adenylyl group from adenosine triphosphate (ATP) to serine, threonine, or tyrosine residues in peptides, altering their function. This post-translational modification is crucial for bacterial processes, including stress response, survival, cell signalling and virulence. Developing targeted therapies, especially against multidrug-resistant ESKAPE bacteria associated with hospital-acquired infection, requires a thorough understanding of this enzyme’s molecular mechanism.

### Structural insights in protein kinases, pseudokinases and protein adenylyltransferase

Structurally, the active site of protein kinases contains a conserved core of a C-terminal and N-terminal convexities referred to as the C-lobe and N-lobe, respectively [62]. The active site is suitable for ATP and metal ion binding, which neutralises the negative charge of the phosphates in ATP [63]. The N-lobe is characterised by having five β-stands, which form an antiparallel β-sheet and an αC-helix. Furthermore, a loop is formed within the N-lobe between the β_1_ and β_2_ strands. This loop contains three glycine residues, termed the glycine rich. It is labelled the G-loop [62]. This loop is critical in the positioning of ATP in the active site [64]. The C-lobe is primarily α-helical and is the substrate binding site [65]. The start of the loop contains an aspartate-phenylalanine-glycine (DFG) motif, where the aspartate binds to metal ions to facilitate the transfer of the γ-phosphate in ATP [66].

**Fig. 1:**
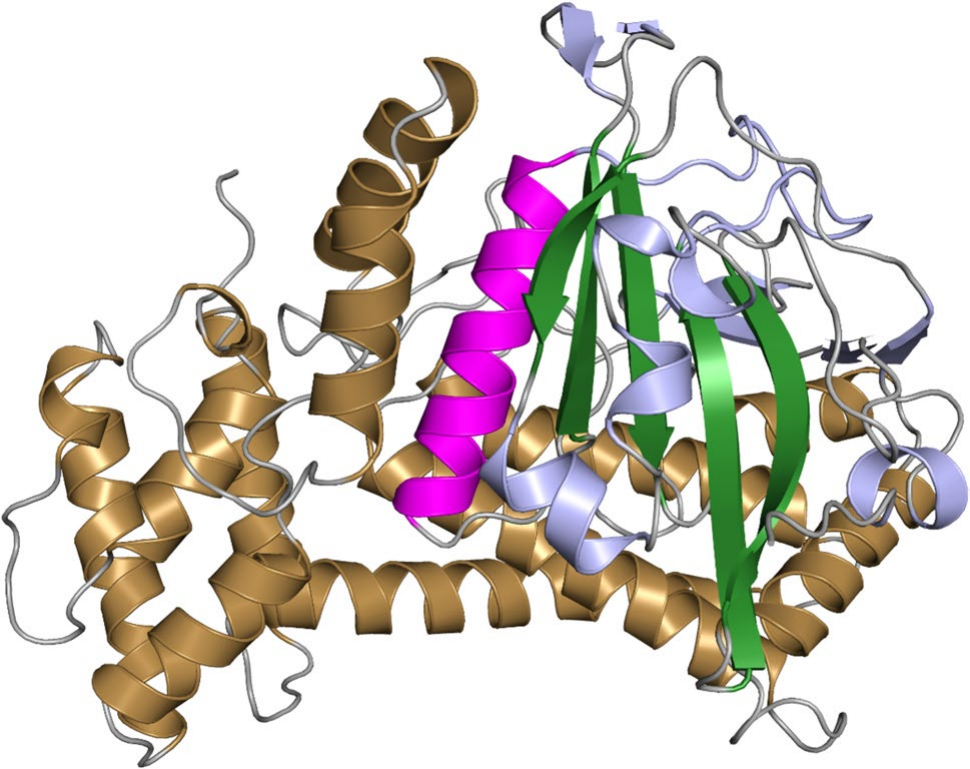
Homology model of *Kp*PrAT. The ribbon structure of the protein (PDB: 6K20) is shown, with the antiparallel β-sheet highlighted in forest green, the αC-helix in magenta, and the C-lobe in sand yellow

Additionally, the activation loop contains serine, threonine, or tyrosine. The catalytic loop contains a histidine/tyrosine-arginine-aspartate (H/YRD) motif and is believed to stabilise the active conformation of the enzyme [67]. Like protein kinases, the domain of pseudokinases has an N-lobe and a C-lobe. The difference is the lack of aspartate in the DFG and H/YRD motifs and the lysine in the β_3_ strand [68]. SelO, a selenoprotein and a pseudokinase, functions in AMPylation in response to oxidative stress [25]. The process involves the transfer of adenosine monophosphate (AMP) from ATP to serine, threonine, and tyrosine residues on protein substrates. An example of a characterised PrAT in ESKAPE bacteria is *K. pneumoniae* PrAT (*Kp*PrAT), which consists of ~ 480 amino acids and has a predicted molecular weight of 54.45 kDa [69]. The homology model was created using Swiss Modelling and based on the *E. coli* PrAT structure (PDB code 6K20), represented as a ribbon structure in Fig. 1.

The solved crystal structure of *Pseudomonas syringae* PrAT bound to a derivative of ATP reveals a protein fold like that of protein kinases, although it lacks the characteristic HRD motif. This structure highlights how PrATs mediate AMPylation instead of phosphorylation, a distinction attributed to the flipped orientation of the ATP derivative within the active site, Consequently, the γ-phosphate, typically involved in phosphorylation, is sequestered within a pocket located between the C- and N-lobes of the protein [25].

### ATP-driven AMPylation by protein adenylyltransferase: mechanisms, and structural adaptations

The α and β-phosphate groups in ATP can be transferred to the hydroxyl groups of amino acid side chains through adenylylation and pyrophosphorylation respectively [70]. Adenylylation or AMPylation, specifically transfers an AMP group to the hydroxyl side chains of serine, threonine, and tyrosine, using ATP as the nucleotide in the reaction. This process results in a substrate containing AMP and pyrophosphate [70, 71]. Bacteria employ AMPylation as a post-translational modification to achieve cellular homeostasis and pathogenicity [72]. Enzymes catalysing adenylylation include the filamentation induced by the cyclic adenosine monophosphate (Fic) family and adenylyltransferase [72]. Adenylyltransferase is part of the nucleotidyl transferase family, characterised by a conserved G-X_11_-D-X-D motif. The aspartate residue within this motif interacts with metal ions in the enzyme’s active site [71].

**Fig. 2:**
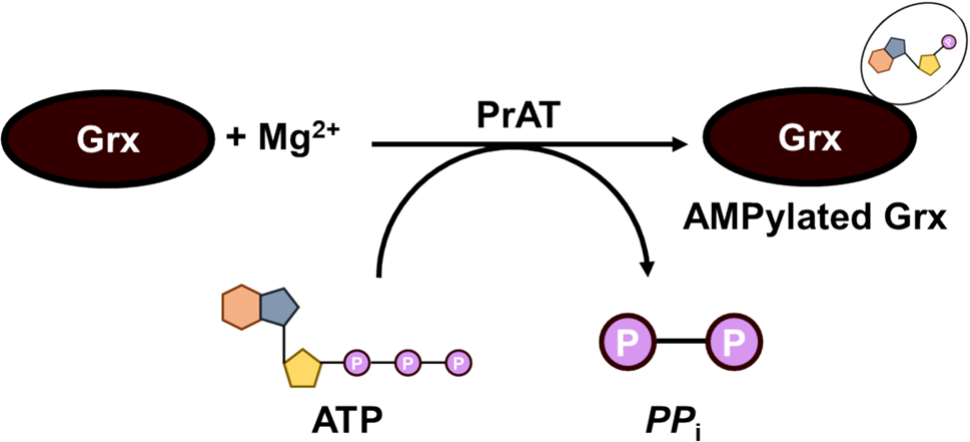
Schematic representation of the reaction catalysed by PrAT. The enzyme catalyses the transfer of an AMP group from ATP, in the presence of Mg^2+^, to Grx. The products of the reaction are an AMPylated Grx and a pyrophosphate

SelO, a protein containing Sec, is predicted to share a structural fold with protein kinases [73] but was classified as a pseudokinase due to the absence of the aspartate residue in the HRD motif of protein kinases [25]. Multiple sequence alignment analysis of bacterial, human, and yeast SelO proteins, compared to protein kinase A (PKA) from *Candida albicans,* reveals the absence of the H/YRD motif in SelO proteins [69]. In bacteria, the Sec residue near SelO’s C-terminus is replaced by cysteine [25]. Despite this, SelO has been found to possess AMPylation activity and is thought to play a role in oxidative stress through its interaction with proteins involved in redox homeostasis [25]. Grx was screened as the potential substrate for PrAT, and the substrate is pivotal in preventing oxidative stress [25, 74]. Additionally, PrAT is a SelO protein and an enzyme that catalyses the transfer of AMP from ATP to Grx, producing an AMPylated Grx and a pyrophosphate, as shown in Fig. 2 [25, 69].

### Role of glutathionylation and deglutathionylation in cellular redox regulation: implications for enzyme activity and the function of PrAT

Glutathionylation involves the formation of a disulfide bond between the thiol group of a cysteine residue in a protein and the thiol group of glutathione (GSH), typically stimulated by oxidative stress because of increased levels of oxidised glutathione (GSSG) [75]. This post-translational modification regulates enzyme activity by either activating or deactivating target proteins. Glutathionylation affects a range of proteins involved in processes such as gene expression, cell signalling, and energy metabolism. Table 1 lists the proteins whose activity is regulated by glutathionylation.

**Table 1:** Proteins that are regulated by glutathionylation

Proteins	Effect of glutathionylation	References
Transcription factors: c-Jun NF-κB	Introduction of the negatively charged GSH moiety. This moiety interacts with the positively charged binding sites on the transcription factor, disrupting its ability to bind to DNA	[76, 77]
Glyceraldehyde-3-phosphate dehydrogenase	oxidation of cysteine 150, a catalytic residue in the protein’s active site, leads to the inactivation of the protein	[78]
cAMP-dependent protein kinase	oxidation of cysteine 199, renders the protein inactive	[79]
Caspase-3	Oxidation of cysteine 135, in the active site, and cysteine 45 leads to protein inactivation	[80]
HIV-1	Oxidation of cysteine 67 increases protein activity, whereas oxidation of cysteine 95 leads to protein inactivation	[81]

**Fig. 3:**
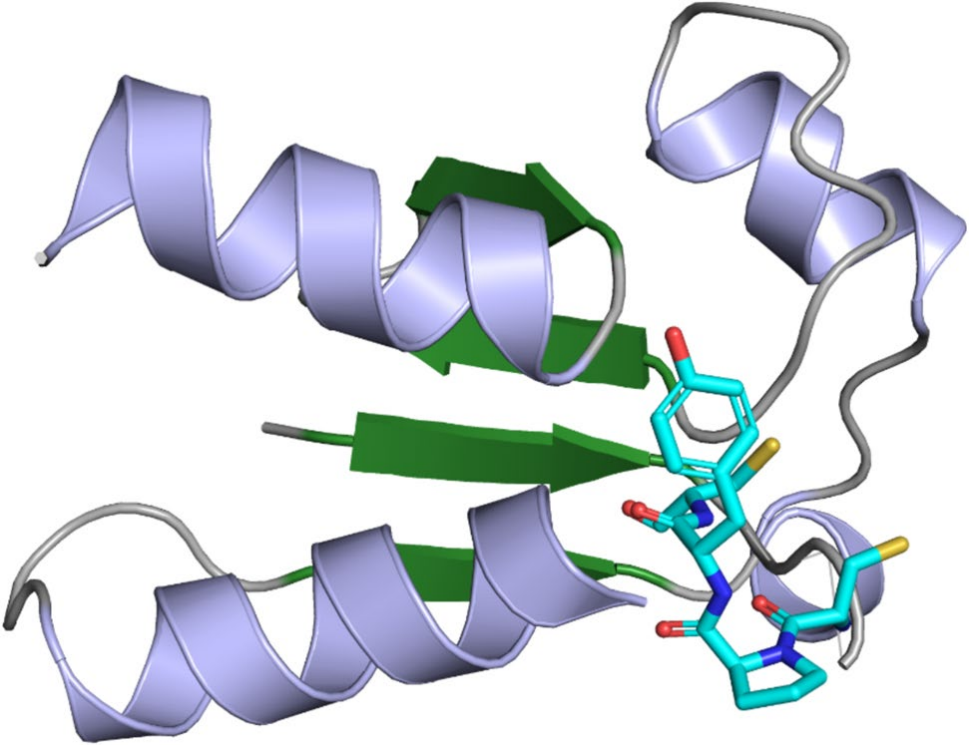
Homology model of *Kp*Grx. The ribbon structure of the protein (PDB:1GRX) is shown, with the 4-β-stranded β-sheet highlighted in forest green and surrounding α-helices in light blue. The active site (CPYC) is depicted as a stick representation, with the sulfur of the thiol side chains of the cysteines coloured in yellow

**Fig. 4:**
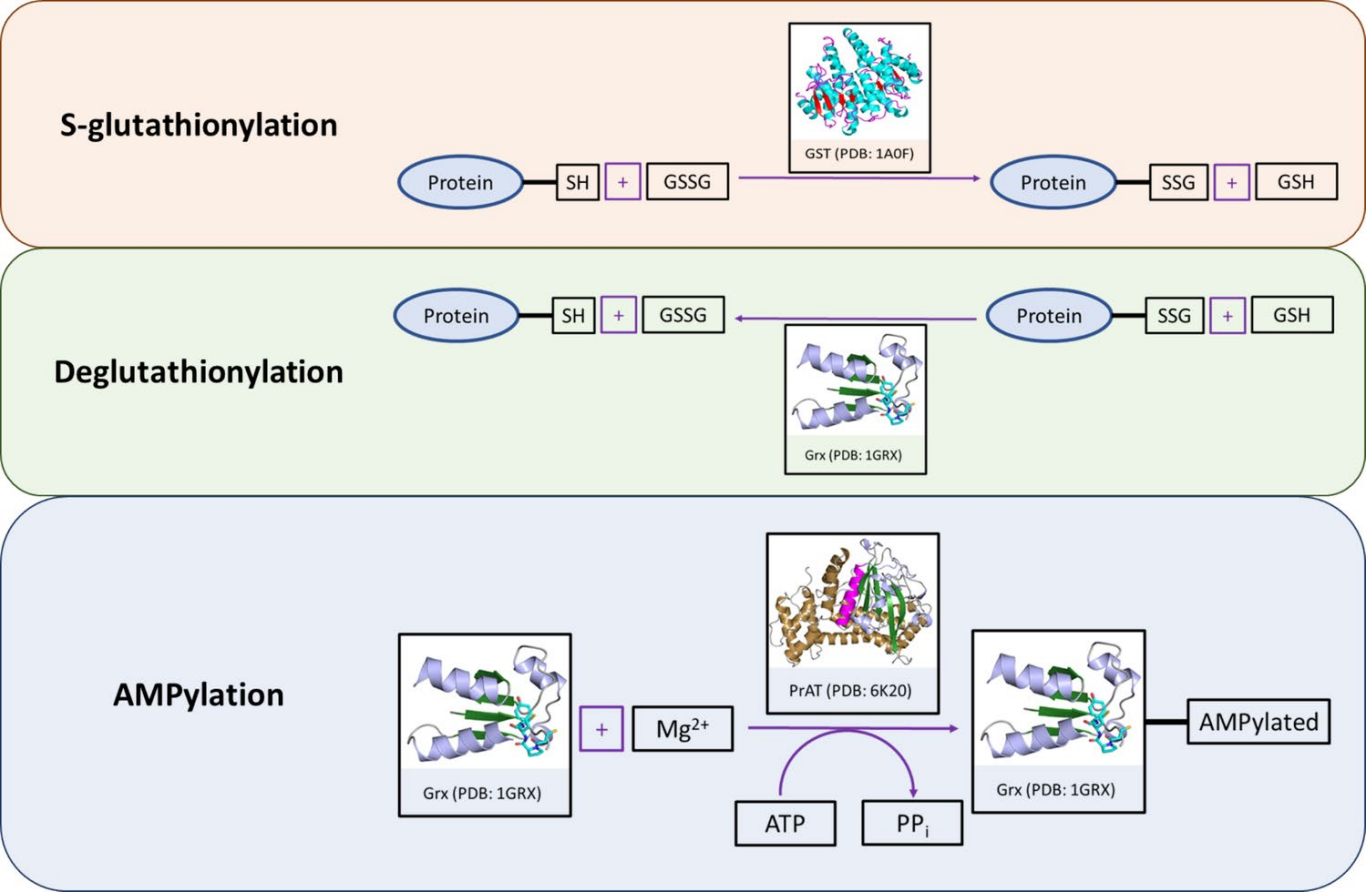
Schematic of the link between the *S*-glutathionylation cycle and the mechanism of PrAT. The forward reaction, known as *S*-glutathionylation*,* involves the conjugation of oxidised glutathione (GSSG) with a protein thiol group, catalysed by glutathione *S*-transferase (GST). This reaction results in the formation of an oxidised protein (Protein-SSG) and reduced glutathione (GSH). The reverse reaction, termed deglutathionylation, is catalysed by glutaredoxin (Grx) and involves the removal of the glutathionyl from the protein, regenerating a reduced protein (Protein-SH) and GSSG. Subsequently, Grx undergoes AMPylation by protein adenylyltransferase (PrAT) in the presence of ATP and magnesium ions

This modification is reversible through deglutathionylation, a process catalysed by Grx, which reduces the protein-GSH mixed disulfide bond, restoring the protein to its native conformation [82]. Interestingly, *E. coli* SelO regulates this process by AMPylating tyrosine-13 in Grx. Grx is a small protein with a molecular weight of ~ 10 kDa [26]. Structurally, it has a fold consisting of a β-sheet with four β-strands, surrounded by three α-helices [83], shown in the ribbon structure of Fig. 3 (PDB: 1GRX). The active site of the peptide contains a CXXC motif, which serves as a GSH binding site [26, 84]. Additionally, Grx is classified into monothiol or dithiol forms based on the number of cysteine thiol groups in the CXXC motif [84]. Oxidoreductases, like Grx are employed to repair oxidised cysteine side chains in proteins. The link between *S*-glutathionylation and AMPylation of Grx is depicted in Fig. 4.

### Distinguishing PrAT from other adenylyltransferases

PrAT is encoded by the *selO* gene, which produces a selenoprotein-O exhibiting adenylyltransferase activity. This enzyme is distinct from the GlnE protein, encoded by the *gln*E gene, also known as glutamine synthetase adenylyltransferase (GS-ATase). In bacteria, GS-ATase has dual functionality: it catalyses the addition of an AMP group to glutamine synthetase (GS), functioning as an adenylyltransferase, and conversely, it can remove the AMP group from GS, acting as a removase [85]. This post-translational modification of GS by GS-ATase plays a crucial role in regulating GS activity, thereby modulating nitrogen assimilation for amino acid biosynthesis in *Mycobacterium tuberculosis* [86]. However, the presence of glutamine and 2-oxoglutarate attenuates the regulatory activity of the GS-ATase in *E. coli* [85].

Beyond PrAT and GS-ATase, several other notable adenylyltransferases contribute to diverse biological processes. These include nicotinate mononucleotide adenylyltransferase (NNAT), involved in nicotinamide adenine dinucleotide (NAD^+^) biosynthesis; aminoglycoside nucleotidyltransferases (ANTs), which confer antibiotic resistance; and phosphopantethiene adenylyltransferase (PPAT), essential in coenzyme A (CoA) biosynthesis [87–89].

## ESKAPE pathogens and protein adenylyltransferase

There are multiple mechanisms that bacteria employ to evade the effects of antibiotics. These mechanisms include decreasing antimicrobial uptake, changing the drug target, inhibiting the activity of the antimicrobial target and producing biofilm [4, 90]. Membrane transporter proteins, or efflux pumps, expel toxic substances, including antibiotics, from bacterial cells, reducing intracellular antibiotic concentrations [91, 92]. In gram-negative bacteria, porins in the outer membrane regulate the entry of hydrophilic substances, further limiting antibiotic uptake [93]. Aminoglycosides disrupt protein synthesis by binding to bacterial ribosomes, but resistance arises through biochemical modifications like acetylation, phosphorylation, and adenylation, which reduce drug-target binding affinity [94–96]. Similarly, β-lactam antibiotics, which target penicillin-binding proteins (PBPs) critical for bacterial cell wall synthesis, are rendered ineffective by β-lactamases that hydrolyse the β-lactam ring [97, 98]. Additionally, biofilms-bacterial communities embedded in a protective polymer matrix-lower bacterial metabolic rates, enhancing survival under hostile conditions and contributing to resistance [90, 99]. The role of protein adenylyltransferase in ESKAPE pathogens remains poorly understood. However, Sreelatha et al. (2018) explored AMPylation activity of SelO in *E. coli*, *Pseudomonas syringae*, a plant pathogen, and *Saccharomyces cerevisiae*. Using a biotinylated ATP analogue and mass spectrophotometry as a comparative technique, the study identified substrates that either interacted with biotinylated AMP or underwent AMPylation during co-expression. Notably, Grx was confirmed as an AMPylation target of *E. coli* SelO. Building on this, Maake and Achilonu (2024) expressed, purified, and biophysically characterised *K. pneumoniae* SelO, revealing its ATP-binding capability. Magnesium ions were found to induce conformational changes that facilitate nucleotide binding.

**Fig. 5:**
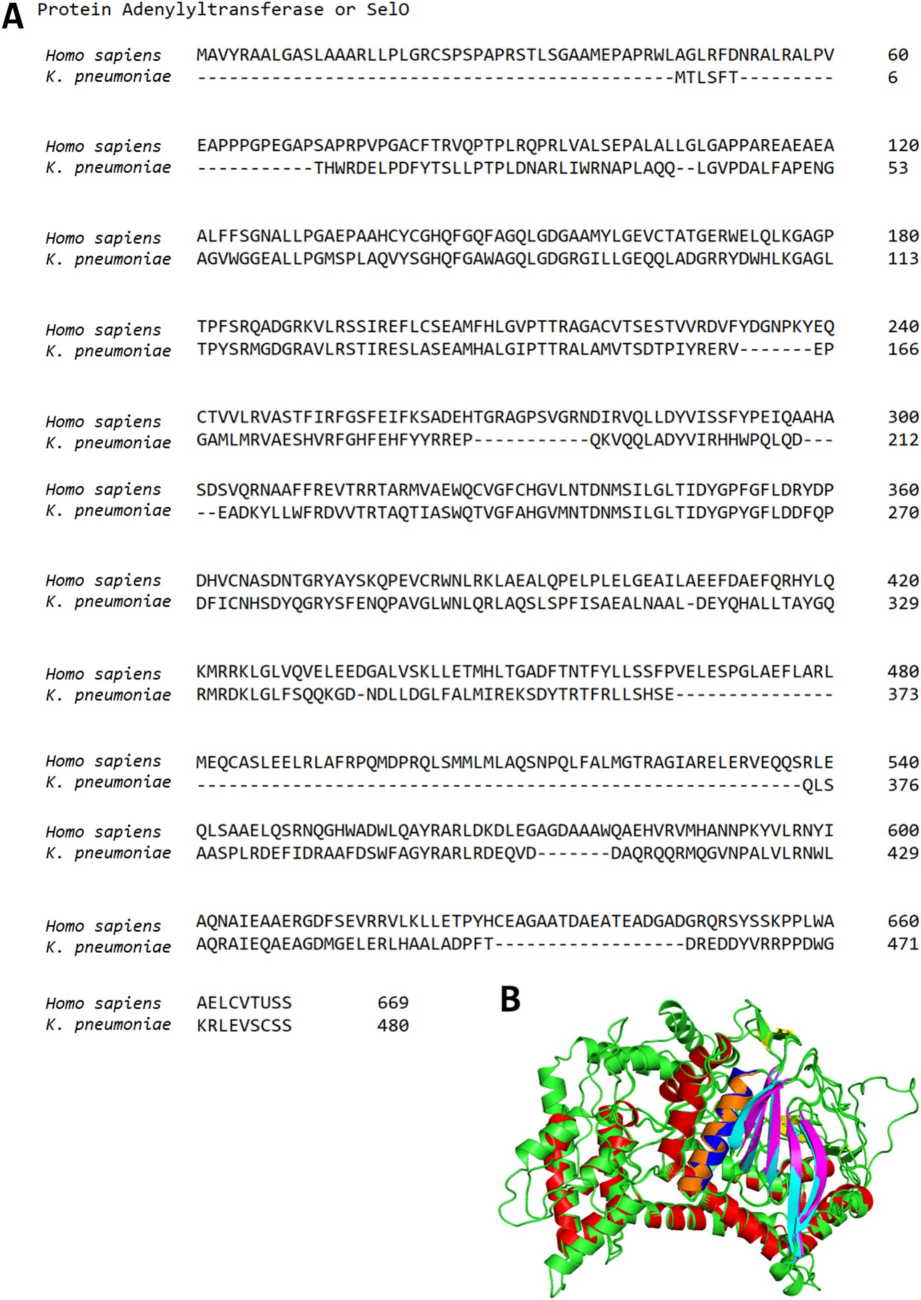
Structural and sequence comparison of human and *K. pneumoniae* protein adenylyltransferase. (A) Sequence alignment of human and *K. pneumoniae* PrATs performed using Clustal Omega. The two pseudokinases share 44.38% sequence similarity. (B) Structural alignment of the AlphaFold homology model of human PrAT (A0A811YKI6.1. A) with the homology model of *Kp*PrAT (6K20), visualised using PyMOL. The C_α_-helix is shown in blue for the human PrAT, and orange for *Kp*PrAT. The five-stranded β-sheet is depicted in cyan for the human PrAT and magenta for *Kp*PrAT

Key Reasons to Target Adenylyltransferase:

1. Essential Role in Bacterial Survival:
  In *P. aeruginosa*, *E. faecium*, and *K. pneumoniae*, adenylyltransferase plays a critical role in the biosynthesis of NAD^+^ biosynthesis, a molecule essential for bacterial survival [100–102]. Inhibiting adenylyltransferases could impair the bacteria’s ability to establish infections and persist in the host.
2. Contribution to Antibiotic Resistance Mechanisms:
  Many ESKAPE pathogens exhibit resistance to aminoglycosides, primarily due to aminoglycoside modifying enzymes (AMEs) that inactivate these antibiotics [103]. Adenylyltransferases, classified as ANTs, are a subgroup of AMEs responsible for this modification. Targeting these enzymes could restore the efficacy of aminoglycosides.
3. Low Homology to human Counterparts:
  The protein sequence of bacterial protein adenylyltransferase shares only 44.38% similarity with its human homologue (Figure 5). This low sequence homology minimises the risk of off-target effects and enhances the specificity of inhibitors.
4. Potential for Novel Drug Development:
  Targeting PrAT represents a relatively unexplored area in antimicrobial drug discovery. Developing inhibitors for these enzymes offers the potential to create first-in-class drugs with novel mechanisms of action, reducing the likelihood of cross-resistance with existing antibiotics.
5. Broad-Spectrum Applicability:
  Protein adenylyltransferase from *E. coli* and *K. pneumoniae* show a sequence similarity of 78%. Furthermore, sequence alignment studies indicate that this enzyme is conserved across multiple ESKAPE pathogens [69]. These findings suggest that inhibitors targeting the enzyme could exhibit broad-spectrum activity, particularly within the ESKAPE pathogens.

These findings underscore the potential of adenylyltransferase as a therapeutic target and offer insights into its biochemical mechanisms in pathogens and model organisms.

## Protein adenylyltransferase (PrAT) as a therapeutic target

There are 25 human selenoprotein-encoding genes, mostly oxidoreductases with Sec residues in their active sites [73]. Among them, SelO is one of the least studied, In mammals, SelO is a mitochondrial protein exceeding 70 kDa [73]. Found in eukaryotes, archaea, and prokaryotes, it contains a conserved CXXU motif, though Sec is replaced by cysteine in some eukaryotes and most prokaryotes [104]. The molecular weight of *K. pneumoniae* SelO is ~ 54 kDa [69], and its sequence shares 44.38% identity with human SelO Fig. 5. As a PrAT protein, SelO interacts with Grx, a redox protein, playing a critical role in oxidative stress, through the modulation of the glutathionylation cycle. Targeting PrAT could exploit oxidative stress to combat MDR bacteria, such as ESKAPE pathogens, in nosocomial infections.

### Evidence supporting protein adenylyltransferase as a viable therapeutic target

In yeast (*S. cerevisiae*), SelO plays a protective role against oxidative stress caused by hydrogen peroxide. Knockout studies demonstrated decreased cell viability under oxidative conditions, while reintroducing wild-type SelO restored it. The AMPylation of Grx in yeast SelO’s active site was linked to regulating glutathionylation [25]. Knockout strains exposed to oxidised GSH showed decreased glutathionylation, suggesting that SelO disruption alters the glutathionylation-deglutathionylation balance, inducing oxidative stress.

PrATs in ESKAPE pathogens possess structurally conserved ATP-binding sites, making them viable targets for competitive inhibitors. Compounds like balanol and H89 bind to the pocket located between the lobes of the conserved catalytic core regions of kinases [105]. STE20-related adaptor (STRAD) is a pseudokinase that forms a complex with tumour suppressor kinase LKB1 and MO25. This complex is essential in the activation of AMPK-related kinases, involved in cellular metabolism and cell cycle regulation. Small Molecule Activators of LKB1 via STRAD (SMALS) enhance the activity of this complex by stabilising it, offering a mechanism of activation without directly targeting the kinase domain [106]. This strategy‒targeting allosteric sites‒offers an alternative when ATP-competitive inhibition is not feasible. Table 2 summarises the potential strategies for designing inhibitors against PrAT.

**Table 2:** Strategies for designing inhibitors against PrAT

Strategy	Description
Grx-mimetic peptide	Peptide that mimics Grx
ATP analogues	Modified nucleotides to block ATP-binding pockets
Allosteric modulators	Compounds binding outside the active site

Figure 5, the multiple sequence alignment of human SelO and *K. pneumoniae* SelO reveals low homology between the two. This reduced similarity reduces the potential of off-target effects, thereby enhancing the selectivity of putative inhibitors. Thus, illustrating the potential for PrAT-targeted treatments that minimise host toxicity.

## Future directions

To advance the therapeutic potential of targeting PrAT, future research should prioritise the identification and characterisation of PrAT homologues in ESKAPE pathogens. While PrAT has been linked to redox regulation and bacterial stress response, its specific structural and functional roles across these pathogens remain underdeveloped. Comparative genomics and functional assays across ESKAPE species may reveal pathogen-specific variations that can be exploited for selective drug development.

Moreover, integrating high-throughput compound screening with structure-based drug design could accelerate the discovery of PrAT inhibitors with narrow-spectrum activity, thereby minimising off-target effects. In silico modelling and mutagenesis studies may also help define the active or allosteric sites unique to PrATs from drug-resistant strains, facilitating the rational design of inhibitors with enhanced specificity and stability under host conditions.

Finally, validating the essentiality of PrAT in vivo‒using knockout models or infection systems‒will be pivotal for establishing its viability as a drug target. These investigations will help clarify the role of PrAT in bacterial physiology and pathogenesis, ultimately informing the development of more precise and effective antimicrobial strategies.

## Conclusion

Nosocomial infections significantly burden healthcare systems, primarily caused by MDR bacteria classified as ESKAPE pathogens, which can evade the biocidal effects of antimicrobial agents. The urgent need for effective therapeutic agents has become apparent due to these pathogens’ emerging resistance to existing antibiotics, mainly stemming from the overuse and improper administration of antibiotics in healthcare and agricultural settings. Targeting PrAT presents a significant therapeutic promise in the fight against infections caused by ESKAPE pathogens. PrAT is an enzyme involved in maintaining redox homeostasis through its association with Grx, an essential redox-regulating protein. Additionally, PrAT plays a critical role in bacterial virulence, and survival making them compelling targets for novel antimicrobial therapies. Although the human homologue of this enzyme remains poorly characterised and exhibits low sequence similarity to its bacterial counterpart, the bacterial PrAT emerges as a compelling therapeutic target. Future research should focus on elucidating the specific role of PrAT in ESKAPE pathogens, as this could be a pivotal step in addressing MDR pathogens associated with nosocomial infections.

## Data Availability

No datasets were generated or analysed during the current study.

## Abbreviations

AMEs: Aminoglycoside modifying enzymes
AMP: Adenosine monophosphate
ANTs: Aminoglycoside nucleotidyltransferases
ATP: Adenosine triphosphate
cAMP: Cyclic adenosine monophosphate
CAUTIs: Catheter-associated urinary tract infections
CLABSIs: Central line-associated bloodstream infections
CoA: Coenzyme A
ESBLs: Extended spectrum β-lactamase
ESKAPE: *Enterococcus faecium, Staphylococcus aureus, Klebsiella pneumoniae, Acinetobacter baumannii, Pseudomonas aeruginosa,* and *Enterobacter species*
Fic: Filamentation induced by the cyclic adenosine monophosphate
Grx: Glutaredoxin
GS-ATase: Glutamine synthetase adenylyltransferase
GSH: Glutathione
GST: Glutathione *S*-transferase
HAIs: Hospital-acquired infections
ICU: Intensive care unit
*Kp*PrAT: *K. pneumoniae* PrAT
PBPs: Penicillin-binding proteins
PKA: Protein kinase A
PPAT: Phosphopantethiene adenylyltransferase
MDR: Multidrug-resistant
MRPA: Multidrug resistant *P. aeruginosa*
MRSA: Methicillin resistant *S. aureus*
NAD^+^: Nicotinamide adenine dinucleotide
Redox: Reduction–oxidation
ROS: Reactive oxygen species
Sec: Selenocysteine
SelO: Selenoprotein-O
SSIs: Surgical site infections
UTIs: Urinary tract infections
VAP: Ventilator-associated pneumonia
VRE: Vancomycin-resistant *Enterococci*
